# An Update on Childhood-Onset Takayasu Arteritis

**DOI:** 10.3389/fped.2022.872313

**Published:** 2022-04-13

**Authors:** Florence A. Aeschlimann, Rae S. M. Yeung, Ronald M. Laxer

**Affiliations:** ^1^Pediatric Immunology, Hematology and Rheumatology Unit, Hôpital Necker-Enfants Malades, Paris, France; ^2^Division of Rheumatology, Department of Pediatrics, The Hospital for Sick Children, Toronto, ON, Canada; ^3^Department of Pediatrics, University of Toronto, Toronto, ON, Canada; ^4^Department of Immunology, University of Toronto, Toronto, ON, Canada; ^5^Department of Medicine, University of Toronto, Toronto, ON, Canada

**Keywords:** Takayasu Arteritis, large vessel vasculitis, childhood vasculitis, pediatrics, review

## Abstract

Takayasu Arteritis (TAK) is a rare large vessel vasculitis affecting the aorta and its major branches. The heterogeneous and often severe clinical manifestations result from systemic and local inflammation as well as end-organ ischemia. Disease flares are common and contribute to accrued damage over time with significant morbidity and mortality. Newer understanding of the pathogenesis in TAK has paved the way for the use of pathway targeting agents such as tumor necrosis factor (TNF)α- or interleuking (IL)-6-inhibitors with improved disease control. Nevertheless, long-term data are lacking, particularly in children; prognosis often remains guarded and the disease burden high. This article aims at providing a comprehensive review of childhood-onset TAK with a focus on recent publications.

## Introduction

Takayasu Arteritis (TAK) is the most common large vessel vasculitis in children. It is characterized by granulomatous inflammation of the aorta, its major branches and the pulmonary arteries that may result in segmental stenosis, occlusion, dilatation and/or aneurysms. Systemic inflammation, local inflammatory processes and organ dysfunction secondary to ischemia lead to a highly variable clinical presentation and significant morbidity if untreated. Therapy is often extrapolated from adult studies given the very low prevalence of the disease in children. Better understanding of pathophysiologic mechanisms has resulted in the use of cytokine targeting therapies. Although early recognition and therapy seem to improve outcome in childhood-onset TAK, long-term follow-up data are lacking and prognosis remains guarded. This article reviews recent publications on epidemiology, pathogenesis, clinical presentation, laboratory biomarkers, imaging, treatment, and outcomes with a focus on the pediatric literature.

## Epidemiology

The incidence and prevalence of TAK are much higher in adults compared to children. A recent systematic review and meta-analysis found an incidence rate of 1.11 per million person-years (95% CI 0.70, 1.76) with considerable variation across different populations (1). Indeed, the described incidence rates of TAK range from 0.3 to 3.3 per million per year and the prevalence from 0.9 to 360 cases per million depending on the population and geographic region studied, with higher rates observed particularly in Asia (2).

Epidemiological data in childhood-onset TAK are scarce. The annual incidence rate reported in a Swedish study was 0.4 (95% CI 0, 1.1) per million for childhood-onset TAK (3). The prevalence estimated from a National Health Insurance database in South Korea varied between 0.04 (95% CI 0.00, 0.08) for children 0 to 4 years old and 0.63 (95% CI 0.36, 0.91) per 100,000 for those 15–19 years old, with an increase of the age-standardized prevalence of TAK over the years (4).

Takayasu Arteritis most commonly affects young women with a peak incidence between 20 and 40 years of age. It is unknown why TAK occurs predominantly in women. In children, female preponderance is lower than in adult-onset TAK and estimated around 2.5:1–3:1 (5). Mean age at disease onset in the pediatric population is 12 years, but TAK has been described across all ages and even in infants (6–9).

## Pathogenesis

While there are still major gaps in our understanding, the etiology of TAK has begun to be elucidated. Current knowledge of pathophysiology is mostly extrapolated from adult studies and animal models of large vessel vasculitis (10). Both, the innate and adaptive immune systems have been implicated in the pathophysiology of TAK (11). Histologically, the inflammatory process usually predominates in the adventitia and the outer part of the media, but may affect all three blood vessel layers. The consequences are vessel wall damage with laminar necrosis and elastic fiber fragmentation, and eventually fibrosis and arterial remodeling (12). Inflammatory cell infiltrates of the arterial wall consist of macrophages and lymphoid cells (αß CD4+ and CD8+ cells, γδ T-cells, natural killer cells, and B cells) (13).

Multiple proinflammatory cytokines have been implicated in the pathogenesis of TAK (14). Increased levels of tumor necrosis factor (TNF)α, interferon (IFN)α, IFNγ, interleukin (IL)-6, IL-8, IL-12, IL-17, and IL-18 have been observed in the peripheral blood of patients with TAK compared to healthy controls. Serum IL-6 and IL-18 levels have been shown to correlate with disease activity (15–18). Novel insights in disease pathways and identification of key pro-inflammatory cytokines have led to the use of cytokine targeting agents such as TNFα- or IL- 6-, and more recently janus kinase (JAK)-inhibitors.

T cells, and particularly Th1 and Th17 responses seem to play an important role in driving the systemic and vascular manifestations in TAK, as demonstrated by increased expression of Th1 and Th17-related cytokines in patients with TAK that correlate with disease activity (17).

Furthermore, recent data indicate the implication of the mammalian target of rapamycin (mTOR) pathway in T cell activation and the development of vascular lesions in TAK. mTOR is a kinase that drives different signaling pathways to regulate cell differentiation, proliferation and metabolism (19), as well as vascular remodeling (20, 21). In addition, mTOR Complex 1 (mTORC1) has been involved in the differentiation of Th1 and Th17 cells (22). In patients with TAK, mTORC1 pathway is hyperactivated in CD4+ T cells and correlates with disease progression (23). This hyperactivity of mTORC1 has been identified as a critical mechanism underlying the altered differentiation of Th1 and Th17 cells (23). *In vitro* and mouse model studies show, that blockade of mTORC1 by sirolimus, a specific mTOR inhibitor, or by genetic knockdown, successfully suppresses the hyperactivation of the mTOR pathway, altered differentiation of CD4+ T cells and arterial inflammation (23, 24). Thus, targeting the mTORC1 pathway may represent an interesting novel therapeutic strategy in patients with TAK (25).

Growing evidence supports the crucial role of the Janus Kinase/Signal Transducers and Activators of Transcription (JAK-STAT) signaling pathway in the pathophysiology of TAK. This was first shown in a large vessel vasculitis mouse-model, in which the JAK1/3 inhibitor tofacitinib significantly reduced vessel wall infiltrates and expression of IFNγ, IL-17, and IL-21, and further diminished angiogenesis and hyperplasia of the intima (26). Using transcriptome analysis, Régnier et al. found an important enrichment for pathways linked to IFN, and especially type I IFN in patients with TAK (27). The upregulation of type I specific IFN gene signature was confirmed in patients with TAK compared to healthy controls, and treatment with either ruxolitinib, a JAK1/2 inhibitor, or tofacitinib reduced T cell activation and restored T cell homeostasis *in vitro* (27). The same group has recently established a specific follicular helper T cell signature that characterizes TAK and highlighted the cooperation of follicular helper T cells and B cells through the JAK/STAT pathway in patients with TAK, further supporting the use of JAK-inhibitors as promising treatment strategy (28). In summary, cytokine signaling dependent on JAK/STAT is critically important in TAK and opens new therapeutic avenues, and studies are currently underway.

The involvement of humoral immune mechanisms has previously been demonstrated by the presence of circulating anti-endothelial cell antibodies (29), autoantibody-producing B cells in inflammatory vascular lesions (30), and increased numbers of plasmablasts in patients with TAK, which correlate with disease activity (31). More recently, the identification of two major endothelial autoantigens, both negative regulators of endothelial activation, and their corresponding autoantibodies further highlighted the involvement of the adaptive immune system in the pathophysiology of TAK (32). These findings supported the use of anti B cell agents in TAK (33).

Furthermore, a complex genetic predisposition may contribute, at least in parts, to the pathogenesis of TAK. Multiple susceptibility loci, including both HLA class I and II, have been identified in various studies, but only HLA-B*52 has been associated with TAK beyond ethnicity (11, 34). Genome-wide association studies established non-HLA susceptibility loci such as *IL12B, IL6, RPS9/LILRB3*, and *FCGR2A/FCGR3A*, which represent potential targets in pathophysiology and treatment of TAK (35).

More recently, monogenic causes including mutations in *NOD2, STAT1* gain-of-function, and *XIAP* have been described in association with TAK (36–39). Clinical features associated with monogenic causes of TAK are early-onset of symptoms, associated other inflammatory and autoimmune diseases such as inflammatory bowel disease or thyroid disorder, and/or features reminding of primary immunodeficiency.

Finally, triggers of the immune response, such as the involvement of Mycobacterium tuberculosis has been suggested for a long time. Although high prevalence of tuberculosis has been described in patients with TAK (i.e., detection of active tuberculosis in up to 18% of patients with TAK, presence of mycobacterium tuberculosis DNA in aortic tissue in up to 70% of cases), particularly in regions with a high prevalence of tuberculosis infection, there is not enough evidence to establish a causal relationship (40). A possible cross-reaction between mycobacterial and arterial antigens is matter of ongoing discussion.

To summarize, almost all arms of the immune response, from cellular components of both the innate and adaptive immune systems to downstream humoral mediators and their signaling components, have been implicated in the pathogenesis of TAK, pointing to the complexity of the immune reaction. It remains to be elucidated, what is causation and what association.

## Diagnosis

The diagnosis of childhood-onset TAK is based on clinical criteria and angiographic abnormalities, and is supported by laboratory findings. It requires a high index of suspicion, especially in children, because (1) onset is often insidious with non-specific symptoms mimicking many inflammatory conditions, (2) the clinical presentation greatly varies due to variable localization and extent of the vessels involved, and (3) the occurrence is rare.

Different sets of classification criteria have been proposed for childhood-onset TAK. The initial 1990 American College of Rheumatology (ACR) classification criteria for TAK were based on data from adult patients with TAK (41). In 2005, the vasculitis working group of the European League against Rheumatism/Pediatric Rheumatology European Society (EULAR/PReS) proposed new classification criteria for childhood-onset TAK (42). These were subsequently endorsed by EULAR, the Pediatric Rheumatology International Trial Organization (PRINTO), and PReS (EULAR/PRINTO/PReS) (43). They optimized the 1990 ACR classification criteria by including the mandatory criterion of angiographic abnormalities (not only conventional angiography, but also more recent imaging modalities such as CT or MRI), as well as the extra criteria arterial hypertension and elevated acute phase reactants. The latter was added to help differentiate TAK from non-inflammatory conditions [e.g., fibromuscular dysplasia (FMD)]. The EULAR/PRINTO/PReS classification criteria for childhood-onset TAK, presented in Table 1, have a sensitivity and specificity of 100 and 99.9%, respectively (43). Of note, these are classification criteria, that are not required to be met to make a diagnosis of TAK and initiate treatment.

**TABLE 1:** EULAR/PRINTO/PReS classification criteria for childhood-onset TAK.

Criterion	Glossary
Angiographic abnormality (mandatory criterion)	Angiography (conventional, CT, or MRI) of the aorta or its main branches and pulmonary arteries showing aneurysm/dilatation, narrowing, occlusion, or thickened arterial wall not due to fibromuscular dysplasia, or similar causes; changes usually focal or segmental
1. Pulse deficit or claudication	Lost/decreased/unequal peripheral artery pulse(s)
	Claudication: focal muscle pain induced by physical activity
2. Blood pressure (BP) discrepancy	Discrepancy of four limb systolic BP > 10 mm Hg difference in any limb
3. Bruits	Audible murmurs or palpable thrills over large arteries
4. Hypertension	Systolic/diastolic BP greater than 95th centile for height
5. Acute phase reactant	Erythrocyte sedimentation rate > 20 mm per first hour or CRP any value above normal (according to the local laboratory)

*CT, computer tomography; CRP, C-reactive protein; EULAR, European League Against Rheumatism; MRI, magnetic resonance imaging; PRES, Pediatric Rheumatology European Society; PRINTO, Pediatric Rheumatology International Trials Organization.*

## Clinical Presentation

Disease often presents with non-specific constitutional symptoms, including fever and systemic inflammation (pre-pulseless stage), and evolves to vascular symptoms attributable to occlusive arteritis. Up to 25% of children are diagnosed during the late inactive, “burnt-out” phase of the disease, where symptoms result from irreversible vascular damage rather than active vasculitis lesions (44–46). TAK may be associated with other inflammatory diseases, such as inflammatory bowel disease, spondyloarthritis or sarcoidosis (47–50). A child with TAK, pyoderma gangrenosum and chronic recurrent multifocal osteomyelitis has also been described (51).

**FIGURE 1:**
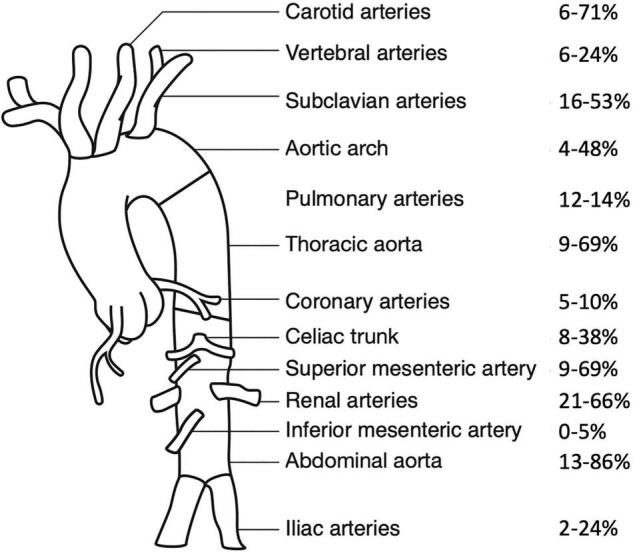
Frequency of arterial involvement at presentation, modified according to Aeschlimann et al. (44). Frequencies of arterial involvement in pediatric Takayasu Arteritis are reported as percentages, paired vessels are presented as one combined value. Data are extracted from Refs. (6, 7, 44, 46, 52, 54, 55, 62).

In children, the aorta (arch, thoracic or abdominal) is most commonly affected, followed by the renal, subclavian, carotid, and splanchnic arteries (5, 52; Figure 1). Stenotic lesions predominate, but occlusion, concentric vessel wall thickening and aneurysms may also be observed. Lesions are characteristically located close to the origin of the aortic branches, with an often segmental and patchy distribution (53).

Table 2 summarizes childhood-onset TAK cohorts published during the last decade (6, 7, 44–46, 52, 54–62). Children may present with non-specific symptoms such as fever, dyspnea, headaches, weight loss or abdominal pain. Unlike adults, musculoskeletal symptoms including arthritis are infrequent (5), although they are more commonly described in pediatric TAK cohorts from South America (54, 56, 63). Cutaneous involvement (erythema nodosum, pyoderma gangrenosum) (51, 64) and ocular disease such as retinal vasculitis (47, 65) are rare in children. Presentation may be dramatic and life-threatening when acute hypertensive crisis, heart failure or arterial dissection develop (66–69).

**TABLE 2:** Demographic and clinical characteristics of pediatric TAK cohorts, modified according to Aeschlimann et al. (135).

Autor (REF)	Zhu (59)	Jales-Neto (54)	Szugye (6)	Goel (55)	Clemente (56)	Misra (57)	Elefthe riou (45)	Feng (58)	Aeschlimann (44)	Sahin (7)	Fan (46)	Lei (60)	Danda (52)	Bolek (61)	Karabacak (62)	Summary	Brunner (136)
Country	China	Brazil	United States	India	Brazil	India	United Kingdom	China	Canada	Turkey	China	China	India	Turkey	Turkey		
Year of publication	2010	2010	2014	2014	2016	2015	2015	2017	2017	2019	2019	2020	2021	2021	2021	2010–2021	2010
Patients (*n*)	14	17	21	40	71	29	11	11	27	16	101	9	119	25	24	535	241
Sex F:M	3.7:1	1.8:1	2.5:1	1.9:1	2.6:1	1.9:1	1.8:1	1.8:1	2.9:1	3:1	3.2:1	8:1	2.4:1	3.2:1	7:1	3.2:1	3.0:1
Age at onset, mean (range), years	10.2 (7–16)	16* (1–18)	11.5 (0.1–17)	12.5* (1–16)	9.2 ± 4.2 SD	13* (IQR 11–15)	11.8 (1–17)	9.4 (1–14)	12.4* (IQR 9.1–14.4) at dx	12.1* (0.5–16.1)	14* (IQR 12–16)	14.3	14* (IQR 11–15)	12.8	14* (IQR 9–14)	12.7	10 (1–18)
**General features, *n* (%)**																	
Fever	4 (29)	7 (41)	3 (14)	18 (45)	NR^d^	16 (55)	4 (36)	5 (45)	5 (19)	7 (44)	13 (13)	4 (44)	35 (29)	NR	6 (25)	127/439 (29)	47/160 (29)
Weight loss	5 (36)	10 (59)	10 (48)	2 (5)	NR^d^	7 (24)^e^	4 (36)	NR	8 (30)	3 (19)	4 (4)	4 (44)	12 (10)	NR	10 (42)	79/428 (18)	44/199 (22)
Headache	9 (64)	8 (47)	3 (14)	21 (53)	NR^d^	6 (21)	4 (36)	3 (27)	9 (33)	6 (38)	1 (1)	NR	37 (31)	NR	10 (42)	117/430 (27)	66/210 (31)
Malaise	NR	NR	NR	21 (53)	NR^d^	7 (24)^e^	NR	NR	13 (48)	8 (50)	9 (9)	4 (44)	40 (34)	NR	NR	102/341 (30)	
Arthritis/arthralgia	6 (43)	7 (41)	3 (14)	1 (3)	46 (65)	4 (14)^e^	1 (9)	NR	NR	7 (44)	2 (2)	NR	NR	NR	NR	77/320 (24)	33/230 (14)
Carotidynia	NR	3 (18)	1 (5)	NR	NR	1 (3)	NR	NR	NR	NR	4 (4)	NR	5 (4)	NR	0	14/311 (5)	
Dyspnea	3 (21)	NR	4 (19)	11 (28)	38 (54)	NR	3 (27)	1 (9)	4 (15)	NR	30 (30)	NR	28 (24)	NR	NR	122/415 (29)	49/210 (23)
Hypertension	13 (93)	11 (65)	12 (57)	29 (73)	60 (85)	22 (76)	8 (73)	11 (100)	15 (56)	10 (63)	71 (70)	3 (33)	79 (66)	13 (52)	13 (54)	370/535 (69)	199/241 (83)
Abdominal pain	NR	5 (29)	2 (10)	9 (23)	NR^d^	NR	1 (9)	4 (36)	4 (15)	5 (31)	4 (4)	NR	13 (11%)	NR	NR	47/363 (13)	33/199 (17)
Syncope	2 (14)	6 (35)	1 (5)	6 (15)	NR^d^	2 (7)	NR	NR	3 (11)	3 (19)	10 (10)	NR	9 (8)	NR	3 (13)	45/408 (11)	4/199 (2)
Skin features	NR	NR	0	3 (8)	NR	4 (14)	1 (9)	1 (9)	NR	NR	NR	NR	NR	NR	NR	9/112 (8)	12/230 (5)
**Organ-specific features, *n* (%)**																	
Decreased pulse	10 (71)	10 (59)	13 (62)	25 (63)	61 (86)	23 (79)	2 (18)	3 (27)	16 (59)	12 (75)	38 (38)	NR	73 (61)	9 (36)^i^	^k^	295/502 (59)	30/230 (13)
Bruits	3 (21)	10 (59)	12 (57)	19 (47)	53 (75)	14 (48)	5 (45)	3 (27)	15 (56)	14 (88)	52 (52)	^n^	55 (46)	NR	^l^	255/477 (53)	38/230 (17)
Claudication	NR	10 (59)	3 (14)	16 (40)	26 (37)	12 (41)	1 (9)	NR	6 (22)	6 (38)	23 (23)	NR	46 (39)	NR	5 (21)^m^	154/476 (32)	32/241 (13)
BP discrepancy	NR	11 (65)	15 (71)	NR	48 (68)	16 (55)	2 (18)	5 (45)	18 (67)	12 (75)	56 (55)	NR	NR	9 (36)	NR	192/329 (58)	NR
Stroke	0	3 (18)	0	3 (8)	NR^d^	2 (7)	2 (18)	NR	3 (11)	2 (13)	6 (6)	0	10 (8)	0	1 (4)	32/453 (7)	39/230 (17)
Cardiac disease	3 (21)^a^	3 (18)^b^	1 (5)	8 (20)^c^	13 (18)^d^	4 (14)^e^	3 (27)^f^	2 (18)	NR	1 (6)^g^	25 (25)^h^	^o^	18 (15)	0	NR	81/475 (17)	52/230 (23)
Ocular disease	3 (21)	5 (29)^b^	2 (10)	7 (18)^c^	15 (21)	6 (21)^e^	1 (9)	NR	4 (15)	2 (13)	38 (38)	NR	14 (12)	NR	NR	97/466 (21)	12/230 (5)^j^

*BP, blood pressure; F, female; IQR, interquartile range; LAD, lymphadenopathy; M, male; NR, not reported; SD, standard deviation.*

*median.

*^a^Chest pain in n = 3 (21%), cardiac murmurs n = 5 (36%), congestive heart failure = 4 (29%), pericardial effusion and cardiomyopathy n = 1 (7%) each. Bruits over subclavian artery and abdominal aorta n = 3 (21%) each.*

*^b^Cardiac disease reported as “myocardial infarction” in n = 1 (6%) and “heart failure” in n = 3 (18%) patients, ocular disease reported as “visual complaints.”*

*^c^Reported as cardiomyopathy and severe aortic regurgitation in n = 1 (3%) patient, ocular disease reported as “visual blurring.”*

*^d^“Constitutional symptoms” (fever, asthenia, and weight loss) in n = 55 (78%) patients, “neurological symptoms” (headache, stroke, syncope) in n = 50 (70%), “gastrointestinal symptoms” (abdominal pain, diarrhea, vomiting) in n = 41 (58%) patients. Cardiac disease reported as “heart failure.”*

*^e^Malaise and weight loss reported as one item, arthralgia/myalgia reported in n = 4 (14%), arthritis in n = 1 (3%) patient, “cardiac disease” reported as congestive heart failure, “ocular disease” reported as “blurring of vision.”*

*^f^“Cardiac disease” reported as ischemic cardiac pain in n = 2 (18%), as cardiomyopathy in n = 3 (27%), as congestive cardiac failure in n = 2 (18%), as valvular heart disease in n = 1 (9%), and as pericarditis in n = 1 (9%) patients.*

*^g^Reported as “heart failure,” and “visual complaints.”*

*^h^“Cardiac disease” was reported as heart failure in n = 25 (25%) and myocardial infarction/ischemia in n = 3 (3%) patients.*

*^i^Numbers combined for decreased pulses and blood pressure discrepancy.*

*^j^Reported as “uveitis.”*

*^k^Pulse loss: carotid 22%, radial 35%, brachial 26%, femoral 35%, dorsalis pedis 26%.*

*^l^Bruits: carotid 39%, subclavian 35%, renal 39%, abdominal aorta 48%.*

*^m^5/ 24 (21) with claudication of upper extremity and 5/ 24 (21%) with claudication of lower extremity.*

*^n^Bruits: carotid 22%, subclavian 22%, abdominal 44%.*

*^o^Cardiac findings: unstable angina 22%, acute myocardial infarction 11%, heart failure 44%, silent 22%.*

Organ-specific manifestations result from ischemia secondary to vascular stenosis. Arterial hypertension is the main presenting feature (56–100% of children depending on ethnicity, with higher prevalence in Asians) and is primarily related to renal artery stenosis and subsequent renovascular hypertension (44, 58). Arterial hypertension and other clinical signs of hypoperfusion such as blood pressure discrepancy between limbs, decreased peripheral pulses and bruits over large arteries are found in over 60% of children at diagnosis and highlight the necessity of a rigorous physical exam, especially in a child with unexplained systemic inflammation. Claudication of extremities, secondary to decreased blood supply, is reported in a third of children at presentation. Abdominal pain, often related to vasculitis of the abdominal aorta or the mesenteric arteries may occur. Prevalence of cardiovascular complications such as cardiomyopathy, ischemic heart disease, heart failure and valvular disease is estimated between 5 and 27% (6, 45), but coronary artery involvement is reported in only about 11% of children with TAK (44). Neurologic involvement including headache, dizziness, seizures, transitory ischemic attacks, and stroke may be observed. In adults, stroke is more often ischemic than hemorrhagic. While ischemic strokes are more commonly a consequence of steno-occlusive lesions in the carotid and vertebrobasilar vessels, hemorrhagic strokes usually occur as a result of steno-occlusive lesions in the abdominal aorta and renal arteries (70).

More recently, several groups have been directly comparing pediatric and adult TAK cohorts (5, 52, 54, 60–62). Childhood-onset TAK has a lower female predominance (5, 52, 54) and a shorter diagnostic delay, possibly due to a more pro-inflammatory presentation (5, 62). Children have more commonly arterial hypertension, less claudication of the upper extremities, and less carotidynia. This reflects the vascular disease pattern, which more often affects the aorta and the infra-diaphragmatic renal and mesenteric arteries in children, but more frequently the aortic arch in adults (5, 52, 61, 62). Higher disease activity scores such as the Indian TAK Clinical Activity Score (ITAS 2010) at diagnosis have been reported in the pediatric compared to the adult population, but this does not seem to be associated with accrued damage over time (52, 54, 62).

Duration of symptoms before treatment initiation has been positively associated with disease extent and damage (52). Disease course is most commonly relapsing-remitting, especially when untreated, but monophasic evolution may be observed.

## Laboratory Features and Serological Biomarkers

Although various biomarkers have been explored for monitoring disease activity in TAK, none has yet been identified for reliable use in clinical practice. In children, elevation of acute phase reactants such as C-reactive protein (CRP) and erythrocyte sedimentation rate (ESR) is commonly observed (45, 55), but their sensitivity to reflect disease activity is uncertain, and they also lack specificity (71). In addition, they rapidly normalize and are masked under IL-6 blockade, adding further challenges to disease activity assessment in tocilizumab-treated patients (72). Anemia and thrombocytosis may be associated with chronic inflammation, particularly in children.

Among inflammatory molecules, IL-6, a pro-inflammatory cytokine that drives CRP synthesis and thrombocytosis and increases ESR seems promising as a biomarker and a treatment target. In tocilizumab-treated patients with TAK, longitudinal IL-6 monitoring might be useful for assessing disease activity and for detecting infections. While infections seem to trigger a significant, but short-term peak in serum IL-6, persistent IL-6 elevation may indicate subclinical disease activity or relapse (73, 74).

Further, IL-6 independent inflammatory biomarkers, such as S100 proteins or pentraxin-3 are being investigated (75). Pentraxin-3, for example, an acute phase protein synthetized locally at sites of inflammation, has been reported to correlate with disease activity in adults with TAK and may help to predict subclinical vascular inflammation (76). As mentioned previously, anti-endothelial antibodies have recently been identified in patients with TAK (32), but like many other serological biomarkers, they have limited availability and are not used in clinical practice; as a result, their clinical value yet remains to be defined. Studies searching for novel laboratory biomarkers are ongoing.

## Imaging

Vascular imaging aims at depicting morphologic anomalies of the aorta and its branches suggestive of arteritis, and at distinguishing active inflammation from chronic inactive disease. Thus, imaging is crucial for diagnosis, assessment of disease extent and follow-up management of TAK. Imaging modalities include conventional angiography, magnetic resonance (MR) angiography, computer tomography (CT) angiography, Doppler ultrasound (US), and fluorodeoxyglucose positron emission tomography (PET) (^18^F-FDG-PET). Although listed in the EULAR/PRINTO/PReS classification criteria for childhood-onset TAK, conventional angiography is nowadays rarely used and restricted to very few, specific indications such as angiographic imaging prior to revascularization procedures (77).

**FIGURE 2:**
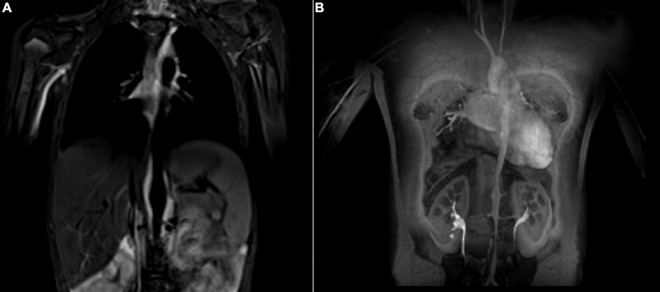
Takayasu Arteritis in a 13-year old girl who presented with fatigue, dyspnea, anemia, and claudication of the lower extremities. The MR angiogram (T2 black blood sequence, coronal view) shows hypersignal (inflammation) of the abdominal aorta **(A)**. Post contrast angiography (coronal view) demonstrates multifocal narrowing of the aorta **(B)**.

In childhood-onset TAK, contrast-enhanced MR angiography is the most popular imaging modality (Figure 2). It is proposed as the first imaging for suspected TAK by the EULAR recommendations on imaging of large vessel vasculitis (77), and is particularly appealing for repeated evaluations in the pediatric population due to lack of invasiveness and of radiation (78). However, recent research evidenced accumulation of gadolinium in the brain and other human tissues with repeated exposure. This raises concerns about potential toxicity particularly in the pediatric population and underlines the importance to carefully consider the indications for each MRI (79). Similar to MR imaging, CT angiography provides information on anatomical changes of the vascular lumen and wall, and the extent of arterial lesions with good spatial resolution. However, radiation exposure remains an important concern, especially in children and with repeated exposure. Doppler US is non-invasive and non-radiating, it visualizes the arterial wall and lumen, as well as altered blood flow characteristics. Challenges with its use include the lack of pediatric radiologists with expertise in vasculitis imaging, as well as acoustic technical limits, restricting the accessibility of certain vessels such as the descending aorta, especially in children (80).

More recently, ^18^F-FDG-PET has been suggested to support radiologic assessment of disease activity (81). In children though, ^18^F-FDG-PET plays a minor role for routine imaging monitoring of disease activity, mainly due to the high radiation dose.

While these imaging modalities (MR angiography, CT angiography, Doppler ultrasound, and ^18^F-FDG-PET) reliably detect signs suggestive of vascular inflammation and allow diagnosis of TAK even in the early pre-stenotic phase, their role for follow-up monitoring is less evident. As an example, vascular changes such as vessel wall thickening or ^18^F-FDG uptake are not specific to active TAK, but may also be induced by healing processes or fibrotic remodeling. This causes difficulties for interpretation of imaging as there is no clear correlation of imaging results with disease activity during the course of disease. Hence, it is crucial to combine clinical, laboratory and imaging evaluation for disease management.

The interested reader is referred to a recent extensive review on imaging in adult and childhood-onset TAK for more information (82).

Efforts to describe angiographic patterns of vascular involvement in TAK have resulted in the identification of different patterns with distinct clinical presentation in various ethnicities (53, 83). Most recently, three distinct clusters with distinct clinical symptoms and outcomes were identified in a large cohort of 806 adults with TAK of Indian and North American origins (84). Yet, these angiography-based disease classifications require validation regarding prognostic prediction.

## Differential Diagnosis

Given the variable clinical presentation with often non-specific symptoms, differential diagnosis of childhood-onset TAK is broad and also depends on disease presentation. For example, differential diagnoses of fever of unknown origin have to be considered during the early disease phase, when non-specific systemic inflammatory symptoms predominate. Infections including tuberculosis and syphilis may cause aortitis, bacterial infections such as *staphylococcus aureus*, *streptococcus*, *salmonella*, or *brucella* should be considered in children with more acute clinical presentations (85, 86).

Differential diagnosis also includes other primary vasculitides (Behçet disease, Kawasaki disease, polyarteritis nodosa) and vasculitides secondary to systemic lupus erythematosus, spondylarthritis or sarcoidosis. Non-inflammatory diseases such as aortic coarctation, Williams syndrome, Marfan or Ehlers-Danlos syndrome, and fibromuscular dysplasia (FMD) may mimic childhood-onset TAK. In contrast to TAK, FMD is not an inflammatory disease; but it may be difficult to differentiate from TAK during the “burned-out” disease phase as–unlike in adults–the characteristic angiographic “string of beads” pattern is rarely observed in childhood-onset FMD (87).

## Treatment

### Immunosuppressive Therapy

Because of progressive or relapsing disease most children with TAK require immunosuppressive therapy to control systemic and vascular inflammation. Challenges in clinical management include the timely treatment initiation and assessment of disease activity to guide therapeutic decision-making. Diagnostic delay can result in irreversible vascular damage even prior to diagnosis and assessment of treatment response is difficult, because reliable laboratory and radiological biomarkers for disease activity are lacking.

Treatment recommendations are extrapolated from adult TAK studies, as high-level evidence including randomized controlled trials is not available to guide treatment in childhood-onset TAK. To date, high-dose corticosteroids remain the mainstay for induction of remission (78, 88), although recent treatment approaches in adults have used protocols without corticosteroids (89). Relapses are common in patients on corticosteroid monotherapy (90), and side effects of long-term high dose corticosteroids may be devastating, especially in children. Therefore, early initiation of second-line, corticosteroid sparing agents has been recommended (78, 88, 91). Traditionally, cyclophosphamide has been used in children with extensive or life-threatening disease, while conventional disease-modifying antirheumatic drugs (DMARDs) such as methotrexate, azathioprine, and mycophenolate mofetil have been favored in less severe cases (45, 91).

Better understanding of disease pathophysiology has resulted in the use of cytokine- and pathway-targeting agents such as TNFα-, IL-6, and more recently JAK-inhibitors (15, 16, 27). Beneficial effects of TNFα- and IL-6-inhibitors have been reported for more than a decade in mainly retrospective case series and case reports. Based on these data, the use of biologic agents on a case-to-case basis was proposed in the recent European consensus-based recommendations for the treatment of childhood vasculitis (78). More recently, and particularly in children, biologic agents have been preferred over cyclophosphamide when they can be accessed and afforded as they present a more favorable toxicity profile.

For TNFα-inhibitors, evidence is mainly extrapolated from adults. The largest cohort study retrospectively included 209 adults with TAK and compared efficacy of TNFα-inhibitors (63% of patients) and the IL-6 inhibitor tocilizumab (37% of patients) (92). Efficacy was equivalent between biologic agents: complete response was observed in 66% of patients on TNFα-inhibitors and 70% of patients on tocilizumab. A total of 103 relapses (median of 36 months follow-up) were reported with similar rates between TNFα-inhibitors and tocilizumab (92). These results are supported by previous studies evaluating the efficacy of TNFα-inhibitors (93–96) and comparing rates of treatment response to TNFα-inhibitors and tocilizumab in patients with TAK (97, 98).

In children, data on TNFα-inhibitors are limited to small retrospective cohorts and case reports. In a retrospective cohort of childhood-onset TAK from Canada, eleven mostly treatment refractory children were treated with TNFα-inhibitors (*n* = 10 treatment episodes) or tocilizumab (*n* = 2 treatment episodes). Biologic agents were associated with significantly better 2-year flare-free survival rates and greater likelihood to reach inactive disease at last follow-up compared with conventional DMARDs or corticosteroids alone (44). Filocamo retrospectively reported four children with mainly refractory TAK treated with TNFα-inhibitors: two achieved remission and two partially responded (99). In addition, several other small case series and case reports have described the beneficial use of TNFα-inhibitors in children with treatment refractory TAK (100–102). TNFα-inhibitors with different mechanisms of action (monoclonal anti-TNFα antibodies and TNFα receptor) have been used, but most commonly the monoclonal anti-TNFα antibody infliximab, followed by adalimumab.

Beneficial effects and safety profiles of the IL-6 inhibitor tocilizumab have been reported in several adult and pediatric TAK cohorts (103–109). Two large retrospective cohort studies of mostly DMARD-refractory adults with TAK demonstrated significantly better event-free survival with tocilizumab compared to conventional DMARDs, and complete response rates in up to 70% of tocilizumab-treated patients (92, 109). However, a randomized, placebo-controlled trial of patients with TAK who had recently relapsed did not find a statistically significant difference between patients receiving tocilizumab and those in the placebo group, although patients receiving tocilizumab trended toward fewer relapses. Among the 36 enrolled patients, six children over the age of 12 years were included (four receiving TCZ, two placebo) and there were no new safety concerns (110). The long-term extension study confirmed a corticosteroid-sparing effect and stable/improved disease on imaging evaluation for up to 96 weeks (111). More recently, a prospective multicenter open-label trial showed high efficacy of tocilizumab in combination with corticosteroids in treatment-naïve patients with TAK, with high remission rates of 85% and corticosteroid discontinuation rates of 54% after 6 months of therapy. However, relapse rates were of 45% after tocilizumab discontinuation, highlighting the necessity of maintenance therapy (112).

In children with TAK, data on tocilizumab are scarce. Apart from the few patients included in the randomized controlled trial (110) there are only a few retrospective case series published, reporting children with TAK with mostly DMARD-refractory disease and good response to tocilizumab with no adverse events (44, 104, 105, 113).

The discovery of the critical role of the JAK/STAT pathway in the pathophysiology of TAK paved the way for the use of JAK-inhibitors. Forty-two mainly refractory patients with TAK treated with a JAK-inhibitor (9/42 treatment-naïve patients, 3/42 with childhood-onset TAK) have been published to date with promising results (27, 114–121). Most patients were treated with the JAK 1/3 inhibitor tofacitinib (39/42) in combination with prednisone ± MTX or other conventional DMARDs. The largest cohort prospectively compared the efficacy and safety of corticosteroids and tofacitinib (*n* = 27 adults with TAK) with corticosteroids and methotrexate (*n* = 26 adults with TAK) (121). Patients receiving tofacitinib had significantly higher complete remission rates at 12 months (88.6 vs. 56.5%, *p* = 0.02), lower relapse rates (11.5 vs. 34.8%, *p* = 0.052) and significantly lower average corticosteroid doses during the study period compared to those on methotrexate. Treatments were well tolerated with a good safety profile (121).

Various other biologic agents have been used with partial success in adults with TAK. Rituximab has been proposed as a therapeutic option following evidence of an implication of B cells in the pathophysiology of TAK (31) and its potential benefits have been reported in retrospective case reports of treatment-refractory adult patients with TAK (31, 33). The use of rituximab in childhood-onset TAK has been described, but sound data are lacking (45). Ustekinumab, a monoclonal antibody against IL-12/IL-23 has been used following detection of *IL12B* as a susceptibility gene for TAK in genome-wide association studies (122). Clinical and laboratory response has been reported to be good, although improvement was not observed on imaging (123). Finally, a randomized, placebo-controlled trial in adult patients with TAK did not find better flare-free survival in patients treated with the T cell co-stimulation inhibitor abatacept compared with controls (124).

However, although biologic agents, and in particular TNFα-, IL- 6-, and JAK-inhibitors seem promising, not all patients respond to these treatments. For anti-TNFα agents, for example, some controversy emerges from reports of patients who developed TAK while they were being treated with a TNFα-inhibitor for another disease and primary non-response or even disease progression has been described under TNFα-, IL-6, and JAK-inhibitors (44, 114, 115, 125–129). Of note, assessment of disease activity is even more challenging in tocilizumab-treated patients, as biologic inflammation may be suppressed and disease activity scores that include acute phase reactants may not be sensitive enough (72, 129).

More data are required to better understand, when to start a biologic agent, which biologic therapy to choose for an individual patient and how long to continue treatment. While biologic agents are currently considered mainly in case of relapsing or refractory disease despite conventional DMARDs, recent treatment approaches have used them at treatment initiation and independently from disease severity in treatment-naïve adults (89, 112) and children (44), but the long-term outcome has yet to be determined. Further studies will be needed to provide more data to guide therapeutic management.

Antiplatelet therapy is often prescribed in the management of TAK, although there is no evidence to support its usefulness. Its benefits need to be weighed against potential side effects, principally gastrointestinal hemorrhage.

### Vascular Interventions

Endovascular interventions or reconstructive surgery may be required to treat major vascular complications. Ideally, they should be performed in phases of stable remission, but urgent interventions may be necessary in case of arterial dissection or critical vascular ischemia (130). In children with TAK, endovascular interventions are performed mainly for treatment-resistant reno-vascular hypertension, restenosis is observed in about half of the patients within one year (131, 132).

## Assessment of Disease Activity and Damage

Assessment of disease activity is often difficult in clinical practice because an outcome measure that reliably reflects vascular inflammation does not exist. The current tools insufficiently reflect disease activity in pediatric large vessel vasculitis, although the Pediatric Vasculitis Activity Score (PVAS) has been validated in children. Other disease activity measurement tools such as the ITAS 2010 and ITAS-A, which additionally includes acute phase reactants, have been developed specifically for TAK, but are only validated in adults.

The US National Institute of Health (NIH) criteria, commonly used to assess disease activity in TAK, define active disease as the presence of constitutional symptoms, new bruits, increased acute phase reactants or new angiographic findings (133).

Although tools for assessment of disease damage exist for adults with TAK, none has been validated in children. These scores may help to assess accumulated damage over time, but discrimination between disease and treatment-related damage may be difficult (134).

## Prognosis/Evolution

Children with TAK present with more systemic inflammation and more widespread vascular disease than adults. However, relapses and accrued damage are high and equally frequent in both groups and seem to be associated with longer duration of symptoms (44, 52, 61).

Recent advances in disease recognition and therapeutic strategies have decreased morbidity and mortality in TAK. Several recent studies have reported the benefit of biologic therapies such as TNFα-inhibitors or Tocilizumab in adults, with up to 80% of patients achieving complete remission at 6 months (92, 112). In children, data are scarce. A retrospective cohort study demonstrated significantly higher 2-year flare-free survival rates and higher rates of inactive disease at last follow-up in children treated with biologic agents, compared with those on non-biologic therapies (44).

Young age and high CRP at disease onset, stroke, lower BMI, longer duration of symptoms and high accrued damage scores have been associated with poor outcomes including increased mortality in childhood-onset TAK (45, 46, 52). In the recent pediatric cohorts, mortality rate varied between 0 and 27% (6, 7, 44–46, 54–59). Despite growing literature, more data are needed, particularly in children, to better describe the long-term outcome of new therapeutic strategies and to identify factors predicting treatment response and relapses.

## Discussion

Timely diagnosis of childhood-onset TAK is important because the disease is associated with a significant morbidity and mortality. Despite persisting gaps in the knowledge of the pathophysiology of TAK, critical inflammatory pathways contributing to the disease begin to be elucidated and provide avenues for new treatment approaches. Treatment recommendations are mostly based on adult TAK studies with level of evidence two or three, although few randomized controlled trials have been published with biologic agents. Corticosteroids remain the mainstay for induction of remission, but biologic agents such as TNFα- or IL-6- and JAK-inhibitors are increasingly used, particularly in severe, relapsing or DMARD-refractory cases. Whether upfront use of biologic agents improve long-term outcome, yet needs to be investigated.

Large international collaborative efforts are required to conduct well-designed studies to determine efficacy of current therapeutic regimens, to identify reliable biomarkers that help to assess disease activity and guide treatment choice, and to better define the long-term outcome of pediatric TAK using validated pediatric outcome measures. One of the main goals is thereby finding therapeutic strategies to reduce cumulative corticosteroid use and to achieve the best possible efficiency-tolerance balance with minimal cumulative damage.

## Author Contributions

FA reviewed the literature and drafted the manuscript. RY and RL reviewed the literature and revised the manuscript critically for important intellectual content. All authors contributed to the article and approved the submitted version.

## Conflict of Interest

The authors declare that the research was conducted in the absence of any commercial or financial relationships that could be construed as a potential conflict of interest.

## Publisher’s Note

All claims expressed in this article are solely those of the authors and do not necessarily represent those of their affiliated organizations, or those of the publisher, the editors and the reviewers. Any product that may be evaluated in this article, or claim that may be made by its manufacturer, is not guaranteed or endorsed by the publisher.
